# The impact of social franchising on the use of reproductive health and family planning services at public commune health stations in Vietnam

**DOI:** 10.1186/1472-6963-10-54

**Published:** 2010-02-28

**Authors:** Anh D Ngo, Dana L Alden, Van Pham, Ha Phan

**Affiliations:** 1 Vietnam Evidence for Health Policy Project, School of Population Health, University of Queensland, Health Strategy and Policy Institute, 138 Giang Vo Str, Hanoi, Vietnam; 2 Shidler College of Business, University of Hawai'i at Mānoa, 2404 Maile Way, Honolulu, HI 96822 USA; 3 Center for Promotion of Advancement of Society, Room 910 - G4 - Trung Yen -Cau Giay, Hanoi, Vietnam

## Abstract

**Background:**

Service franchising is a business model that involves building a network of outlets (franchisees) that are locally owned, but act in coordinated manner with the guidance of a central headquarters (franchisor). The franchisor maintains quality standards, provides managerial training, conducts centralized purchasing and promotes a common brand. Research indicates that franchising private reproductive health and family planning (RHFP) services in developing countries improves quality and utilization. However, there is very little evidence that franchising improves RHFP services delivered through community-based public health clinics. This study evaluates behavioral outcomes associated with a new approach - the Government Social Franchise (GSF) model - developed to improve RHFP service quality and capacity in Vietnam's commune health stations (CHSs).

**Methods:**

The project involved networking and branding 36 commune health station (CHS) clinics in two central provinces of Da Nang and Khanh Hoa, Vietnam. A quasi-experimental design with 36 control CHSs assessed GSF model effects on client use as measured by: 1) clinic-reported client volume; 2) the proportion of self-reported RHFP service users at participating CHS clinics over the total sample of respondents; and 3) self-reported RHFP service use frequency. Monthly clinic records were analyzed. In addition, household surveys of 1,181 CHS users and potential users were conducted prior to launch and then 6 and 12 months after implementing the GSF network. Regression analyses controlled for baseline differences between intervention and control groups.

**Results:**

CHS franchise membership was significantly associated with a 40% plus increase in clinic-reported client volumes for both reproductive and general health services. A 45% increase in clinic-reported family planning service clients related to GSF membership was marginally significant (p = 0.05). Self-reported frequency of RHFP service use increased by 20% from the baseline survey to the 12 month post-launch survey (p < 0.05). However, changes in self-reported usage rate were not significantly associated with franchise membership (p = 0.15).

**Conclusions:**

This study provides preliminary evidence regarding the ability of the Government Social Franchise model to increase use of reproductive health and family planning service in smaller public sector clinics. Further investigations, including assessment of health outcomes associated with increased use of GSF services and cost-effectiveness of the model, are required to better delineate the effectiveness and limitations of franchising RHFP services in the public health system in Vietnam and other developing countries.

## Background

Franchising of health facilities has improved provision and increased use of RHFP services in several low- and middle-income countries [1-5]. Social franchising employs a high quality, client-focused and branded "business model" designed to improve perceptions of service quality and satisfaction. Implementation of social franchising involves three strategic initiatives: improving service quality, increasing service availability and actively promoting the new franchise brand [2]. The combination of improved quality and new services is expected to increase service use. This study analyzes behavioral outcomes associated with a new approach - the Government Social Franchise (GSF) model - designed specifically for application in Vietnam's community-level public health system (CHSs) using a quasi-experimental design that compared franchised with non-franchised health stations.

Since the introduction of *Doi Moi*, which translates as "Renovation" and refers to economic liberalization initiated by the Vietnamese government in 1986, movement towards an "open" economy has had major impacts on the nation's health system. For example, the government removed a longtime ban on privatization of healthcare, allowing a public-private mix. As a result, the number of private providers has grown, increasing competition with the public health system [6]. In addition, the Vietnam government began to generate public sector revenue by introducing user fees for healthcare services that were previously free. In addition, restrictions on patients seeking care at higher-level public hospitals (e.g., tertiary hospitals) without referral from lower level health facilities were removed. As a result, patients have more health service choices at all levels provided they can pay service fees.

The CHS is the primary unit of the public health care system in Vietnam. It is responsible for preventive and curative health care including RHFP services. While user fees are formally applied at the district, provincial, and central hospitals, primary health care services provided at the local CHS are still subsidized or partially subsidized by the government. In addition, the national population program is based in the local CHS and provides free FP services (e.g., condoms, contraceptive pills or injection, intra-uterine device). Given scarce resources, CHS ability to invest in infrastructure development and training is limited. As a result, service quality perceptions have been negatively affected by concerns about staff qualifications, outdated equipment, and limited drugs and supplies [7]. This, in turn, has led many patients to seek care at private clinics or bypass their local CHS and visit higher level hospitals even for minor health problems. Two national living standards surveys in 2004 and 2006 reported that the proportions of out-patient treatment were lowest in the CHS system (22.1% in 2004, 23.4% in 2006) compared to government hospitals (25.2% in 2004, 29.9% in 2006) or private clinics (42.8% in 2004, 34.6% in 2006) [8]. Self-treatment using medicine purchased from a pharmacy is also common [9]. All of these factors have resulted in low use of family planning, antenatal and delivery services through the CHS network [10].

While the stations' primary responsibility is to provide free primary health care services, the clinics are not restricted from charging fees for specialized services or services higher in quality than mandated by the government. However, few CHSs have established market-oriented businesses that generate revenue from user fees for specialized or high quality services (e.g., ultrasound test services). In addition, CHS user fees are not formalized or standardized.

The government of Vietnam has made concerted efforts to strengthen the CHS system's offering of low-cost, high quality RHFP services in order to meet the targets specified in the "10-year National Strategy for Reproductive Health (2001-2010)" and to ensure universal access to quality RHFP services throughout the country. CHS staff (e.g., midwives) are occasionally provided post-graduate training on RHFP services, updating clinical knowledge and skills regarding diagnosis and management of RH problems, e.g., reproductive tract infections, pre- and post-natal care, and other maternal and child health problems. However, the training primarily focuses on clinical and technical aspects of health services with little attention on service provider attitudes and promotional techniques.

In an effort to strengthen the reproductive health capacity of the CHS system, the Provincial Health Departments in Da Nang City and Khanh Hoa Province, funded by a private foundation and technically assisted by Marie Stopes International Vietnam, developed and tested a new social franchise model that involved networking and branding public rather than private clinics. While franchising private RHFP services has been found to improve service quality and use in developing countries [1,4], there is very little evidence to demonstrate that franchising RHFP services improves service capacity in public health clinics. The *Gold Star Project *in Egypt (RHO Archives 2005) is an exception but involved a very different culture (Middle Eastern Muslim) and full rather than partial franchising [11]. The model tested in Vietnam emphasized franchising RHFP within a set of broader clinic services (partial franchising). Thus, the ultimate goal of the field study was to determine whether the social franchise approach, which has had great success in the private sector [1,3-5], could be effectively adapted for use in the public sector using an approach referred to as the Government Social Franchise (GSF) model. GSF membership is hypothesized to increase client service use of franchised CHS clinics compared to non-franchised control clinics.

## Methods

### Study setting

Da Nang is a major city in central Vietnam with 0.8 million residents [12]. Recently, the city has experienced rapid economic development and urbanization. The public health system includes 7 provincial hospitals, 6 district hospitals and a network of 56 CHSs. In addition, there are 4 private hospitals and more than 1000 registered private clinics. Khanh Hoa province is situated in south central Vietnam with a population of 1.16 million [12]. The province has one city, a smaller town, and large rural and mountainous areas. Most people live in rural areas. The public health system has 5 provincial hospitals, 8 district hospitals, and 135 CHSs. There is no private hospital in the province, but there are 300 registered private clinics. At higher levels of the state health system, RHFP services are available at district health centers and the provincial general hospital. In addition, the provincial center for reproductive health care provides RHFP services and carries out communication programs to promote access.

### Description of GSF intervention

The GSF model is described in detail elsewhere [13]. In this initial test of the model, ten CHS clinics in Da Nang and 28 in Khanh Hoa joined the social franchise network. In recruiting CHS clinics to the franchise network, care was taken to create a sample that was generally representative of their respective provinces. Prior to launching the network in July, 2007, GSF network staff received extensive training on: customer relationship management; service quality evaluation; financial sustainability; social marketing and branding; and clinical instruction on RHFP service delivery. CHS doctors and midwives received additional training on quality of care and clinical service delivery. Training was provided by experts recruited and managed by Marie Stopes International Vietnam.

Employing the partial franchising approach, RHFP services in member clinics were branded separately from other CHS services under a new name, *Tinh chi em *(Sisterhood). Participating clinics were required to meet quality standards regarding service delivery, clinic facilities and appearance, service quality assurance, and measurement/evaluation in order to join the GSF network and display the *Tinh chi em *brand. The franchised CHSs were also required to carry out communication activities to promote the new brand. User fees for franchised RHFP services were standardized across the network.

Extensive external marketing activities including road shows, media tours of the social franchise network and dissemination of print media (e.g., brochures, leaflets, banners, local newspaper articles) increased interest in the new brand. A professional marketing company was contracted to design and implement these activities following consultation with CHSs and target groups (e.g., women at reproductive ages). Two commune mass organizations (i.e., *Women's Union *and *Youth's Union*) were mobilized to collaborate with CHSs to maintain marketing and communication activities based in both the CHS and community after the launch. These marketing efforts were designed to look and feel similar to cultural and social events in which Vietnamese women and their families already participated. In addition, two paid "brand ambassadors" (from the *Women's Union *and the *Youth Union*) were recruited and trained by experts in all communes with a CHS franchise member. All brand ambassadors had experience working with other community health programs. They used face-to-face communications to encourage targeted segments (i.e., women, their partners and other family members) to visit and refer others to the GSF clinics. Brand ambassadors were also connected to the community through their affiliation with local mass organizations. Such connections helped build direct brand communication channels and establish a referral network.

### Evaluation design

Assessing the effectiveness of the GSF model, this study was designed to measure the extent to which use of RHFP and other health services at 38 franchised clinics in Da Nang and Khanh Hoa increased over a one year period following launch of the franchise (i.e., July, 2007- July, 2008), However, 2 clinics were removed from the GSF network during the study period because they were unable to uphold service quality standards (e.g., maintenance of physical appearance and standard clinical procedures) and continue communication activities, and did not apply standardized fees for services. This resulted in a final sample of 36 GSF clinics for the study.

A quasi-experimental design with pre-test (baseline just prior to the GSF launch) and post-test (one year following launch), with a control group was employed. To avoid contamination, 36 CHSs recruited in two neighbor provinces (Quang Nam and Phu Yen) were selected as controls using four criteria: 1) availability of a medical doctor, 2) urban or rural geographic location, 3) recent infrastructure upgrades, and 4) availability of RHFP services. The two comparison provinces are fairly similar to the intervention provinces in terms of social and cultural characteristic although per capita income is lower in the control groups [12]. Other characteristics of the CHSs and communes that could affect service use were identified and measured including: distance from the commune to the provincial RH care center or general hospital, number of reproductive age women, and population in the commune, availability of specialized services other than primary health care, and availability of government health insurance.

Given the percentage of users found in the pre-intervention surveys, (37% in Da Nang and 44% in Khanh Hoa), sample sizes of 673 users/non-users in Da Nang and Quang Nam and 508 in Khanh Hoa and Phu Yen had a statistical power of 80% to detect a 20% increase in the proportion of users relative to the total population of reproductive age men and women. Since the majority of RHFP service users are female, males were under-sampled (20%). Two-stage cluster sampling was applied to recruit a demographically representative sample of users/non-users. Seven final stage clusters were selected in Da Nang and Quang Nam, and 5 in Khanh Hoa and Phu Yen. All households in selected clusters were contacted and invited to participate. The study was approved by the Department of Health ethics review committee in each province.

### Data and measurement

Assessing the use of RHFP services in association with the franchise model focused on three outcome measures as dependent variables: 1) client volumes over a 12 month period as reported by study CHSs; 2) self-reported usage rates (the proportion of self-reported RHFP service users at participating CHSs over the total sample of respondents); and 3) self-reported RHFP usage frequency (the number of visits for RHFP services at the local CHS) at study clinics over a 12 month period.

To collect data on CHS client visits, a reporting system was developed by the research team. A reporting form was designed to be uniformly used in both franchised and control CHSs. Each month, the CHS report on client visits was submitted to the provincial department of health before it was sent to the research team. To ensure accuracy of the data, client reporting was monitored by a field supervisor appointed by the provincial department of health (for control provinces) and the M&E team (for intervention provinces) through regular visits to the clinics and review of reported data that was then double checked by the research team.

Usage rates and clinic visit frequency in the previous 12 months were self-reported in household surveys of users and potential users conducted just prior to launch (baseline) and again at 6 months and 12 months following implementation of the GSF model. In each sampled cluster, trained surveyors interviewed all household members aged 15-49, either 5-8 P.M. on weekdays or during the day on the weekends. All males and females were interviewed in 6 of 12 groups of GSF and control communes. Only females were interviewed in the other 6 groups. A list of respondents and their home addresses compiled from the baseline survey was used to contact and make appointments with respondents for follow-up interviews in the two subsequent surveys. At baseline (pre-GSF launch), respondents were asked whether they had visited their local CHS in the past 12 months and recount the number of visits for each specific RHFP service (e.g., condom, Gyn/Ob checks up, delivery, IUD insertion) listed in the questionnaire. Those who reported using any RHFP service at their local CHS were defined as users, otherwise non-users. In the two follow up surveys, respondents were asked whether they had visited their local CHS for health services in the past 6 months since the previous survey. They were also asked to recount the number of visits to their local CHS for each specific RHFP service. Verbal consent was obtained before each interview. Each participant was paid VND 20,000 (US$ 1.2) for his or her time.

### Data analyses

Data analyses were undertaken in two sequential steps. First, descriptive statistics on each outcome measure at both intervention and control group were performed. In each study group, baseline and follow up data were compared using chi-square test for usage rate as a dichotomized variable, pair-*t *test for CHS client volumes and self-reported service use frequency as continuous variables. The second step involved regression analyses to test for an association between franchise membership (franchised versus non-franchised) as the key independent variable and each of 3 outcome measures listed earlier (as the dependent variable). The regression analyses also controlled for baseline differences between the two study groups.

Treating CHS as the unit of analysis, the first regression model featured the log of the volume of clients (as reported by the clinics) for 12 months following launch of the GSF network as the dependent variable. In addition to franchise membership, independent variables included: 1) distance from the commune to the provincial RH care center or general hospital; 2) availability of ultrasound service; 3) the log of numbers of reproductive age women or total population in the commune.

Taking individual respondent as the unit of analysis, to assess usage rates, a logistic regression model was run with the proportion of respondents who self-reported visiting the local CHS for any RHFP service (yes/no) as the dependent variable. Independent predictors other than franchised versus non-franchised were: respondent demographic characteristics, and clinic-/cluster-level correlated responses. Finally, a third regression model tested hypothesized associations between self-reported frequency of service use in the past 12 months (dependent variable) and franchise membership (independent variable), controlling for differences between respondents in intervention and control communes.

## Results

### Descriptive statistics

Table 1 displays baseline characteristics of the 72 (36 GSF and 36 control) CHSs employed in our evaluation. Table 2 presents baseline demographics of surveyed respondents. The initial baseline survey sampled 1,181 individuals in each group of GSF and control clinics (a total of 2,362 respondents). In the 6 month follow up survey, 90% (91%) of baseline (control) group respondents participated. In the final survey (1 year from baseline), response rates were 82% in franchised communes and 80% in control communes. Although, there were statistically significant differences across the two groups, inclusion of the demographic variables in the regression models controlled for possible confounds.

**Table 1:** Baseline characteristics of sampled CHSs

Variable	Intervention (n = 36)		Control (n = 36)	
	
	n	%	n	%
Location**				

Urban	14	39	14	39

Rural	22	61	22	61

Having a doctor**	26	72	26	72

Being upgraded**	36	100	36	100

**Distance to the provincial hospital or Center for RH Care **(*p *= 0.52)				

5 km or less	10	28	8	23

5<distance≤20 km	13	36	10	27

More than 20 km	13	36	18	50

Not providing delivery service**	6	17	6	17

Having ultrasound test service* (*p *= 0.054)	5	14	0	0

Implementing health insurance **	36	100	36	100

*Significant difference between intervention and control group (*p *< 0.05).

**Control selection variable.

**Table 2:** Baseline characteristics of surveyed respondents

Variable	Intervention (n = 1066)		Control (n = 1077)			Variable	Intervention (n = 1066)		Control (n = 1077)	
			
	n	%	n	%			n	%	n	%
**Gender **(*p *= 0.34)						Religion*(*p *= 0.00)				

Male	127	11.9	143	13.3		Buddhism	497	46.6	167	15.5

Female	939	88.1	934	86.7		Protestant	0	0	48	4.5

Age* (years) (*p *= 0.01)						Catholic	103	9.7	19	1.8

15-19	133	12.5	83	7.7		Praying ancestors	67	6.3	168	15.6

20-29	272	25.5	256	23.8		Non-religion	398	37.3	668	62

30-39	374	35.1	434	40.3		Other	1	0.1	7	0.6

40-49	287	26.9	304	28.2		Education*(*p *= 0.00)				

Marital status* (*p *= 0.00)						Secondary or less	667	62.6	766	71.1

Single	268	25.1	148	13.7		High or more	399	37.4	311	28.9

Other	798	74.9	929	86.3		Occupation*(*p *= 0.00)				

Number of children* (*p *= 0.00)						Farmer/Fishery	161	15.1	430	39.3

None	236	22.1	98	9.1		Student	134	12.6	79	7.3

1-2	539	50.6	658	61.1		Housewife/unemployed	399	37.4	302	28.0

More than 2	291	27.3	321	29.8		Other	372	34.9	266	24.7

						**Residential location **(*p *= 0.38)				

						Urban	355	33.3	378	35.0

						Rural	711	66.7	699	65.0

*Statistically significant difference between intervention and control (*p *< 0.05).

Table 3. summarizes outcome measures at baseline and 12 months after formal launch of the GSF model for both the intervention and control groups. Generally, client volumes increased significantly for the two CHS groups from the 12 month pre-launch measurement period to the 12 month post-launch period (*t*-test: *p *< 0.05). For example, the number of client visits for RH services in franchised (non-franchised) clinics increased from 1,368 (1,937) at baseline to 2,757 (3,105) at the end of first 12 month post-launch period. The percentage of respondents reporting use of RHFP services at their local CHS during the past 12 months in GSF communes increased significantly from 39% at baseline to 51% (Chi-square test: *p *= 0.000). In the control communes, the corresponding growth in reported use was not significantly different (51% at baseline, and 55% a year later; Chi-square test: *p *= 0.09). The mean number of self-reported visits for RHFP services in the past 12 months to franchised clinics increased significantly from 2.17 at baseline to 2.83 (*t*-test: *p *= 0.008). That same statistic decreased significantly in the control clinics from 2.20 to 1.79 (*t*-test: *p *= 0.000).

**Table 3:** Descriptive statistics of 3 outcome measures at baseline and 12-month follow up

Outcome measure	Intervention			Control		
	
	Baseline	Follow up	*p*	Baseline	Follow up	*p*
Average number of client visits per CHS						

RH	1,368	2,757	0.000*	1,937	3,105	0.000*

FP	962	2,049	0.000*	1,601	1,964	0.048*

All services	6,061	7,310	0.006*	6,614	6,149	0.800

Usage rate (%)	39	51	0.000*	51	55	0.090

Average number RHFP visits per client	2.17	2.83	0.008*	2.2	1.79*	0.000*

*: Statistically significant difference between baseline and follow up data (*p *< 0.05)

### Results of regression analyses

Table 4 presents the results of the regression analyses (regression coefficient [coeff] and 95% confidence interval [CI]) modeling association between franchise membership and client turnover 12 months following implementation of the GSF program. Compared to the controls, GSF network clinics experienced higher user volumes overall and for reproductive health services in particular (total client volume coeff = 0.35, 95% CI = 0.11, 0.59; RH volume coeff = 0.41, 95% CI = 0.07, 0.76; p < 0.05). The increase in family planning service client volume was marginally significant (FP client volume coeff = 0.37, 95% CI = 0.00, 0.73, p = 0.05). Calculated as the antilog of the coefficients of the logged outcomes, after controlling for other independent variables and baseline differences, franchise membership was associated with a 40% increase in total use; a 51% increase in RH use; and a 45% increase in FP use. Other CHS characteristics were not significantly associated with the client volume.

**Table 4:** Association between franchise membership and client turnovers in past 12 months

	Log of RH client [Coeff (95% CIs)]	Log of FP clients [Coeff (95% CIs)]	Log of all services clients [Coeff (95% CIs)]
**Intervention status**			

Non-franchised CHS	Ref	Ref	Ref

Franchised CHS	0.41(0.06-0.76)*	0.37(0.00,0.73)*	0.35(0.11,0.59)*

**Distance to the provincial hospital or Center for RH Care**			

5 km or less	Ref	Ref	Ref

5<distance≤20 km	-0.29(-0.41,0.35)	0.19(-0.24,0.62)	0.62(-0.23,0.35)

More than 20 km	0.17(-0.19, 0.53)	0.10(-0.29,0.50)	0.01(-0.26,0.28)

**Log of numbers of women/Population**	0.04(-0.29,0.37)	0.16(-0.19,0.52)	0.06(-0.21,0.34)

**Having Ultrasound test**			

No	Ref	Ref	Ref

Yes	-0.15(-0.74,0.45)	-0.08(-0.74,0.57)	-0.23(-0.66,0.20)

*Statistically significant (*p *< 0.05).

Looking at the results of the logistic regression model, after 12 months, the association between the probability of self-reported use and franchise membership was not statistically significant (OR = 1.34; *p *= 0.15). Among other covariates, the logistic regression analysis found that non-farmers were significantly less likely than farmers to visit their local CHS for RHFP services (OR = 0.58; *p *= 0.000) and ever-married individuals were more likely than those who were single to use RHFP services provided at their local CHSs (OR = 2.1; *p *= 0.004)

With regards to client self-reported frequency of RHFP service use, the regression model shows that franchise membership is associated with a significant increase in the average self-reported frequency of client visits during the test period (self-reported visit frequency coefficient = 1.20; p = 0.000). With the exception of Buddhist versus Protestant Christian self-reported affiliation, other covariates were not significantly associated with CHS visit frequency.

## Discussion

Overall, this study indicates that the GSF model has the potential to significantly increase RHFP service use at local public health clinics in Vietnam. Increased client use (as indicated by clinic records) was consistent with increased self-reported visit frequency. The 40% plus increase in RH clients and total clients as well as the 20% increase (Coeff = 1.20) in the frequency of self-reported visits in the previous 12 months are substantial. However, the absence of statistical evidence indicating a positive association between self-reported usage rates and franchise membership suggests that significantly higher client volumes reported by the franchised clinics may have resulted from increased visit frequency by existing clients, not from new clients.

Even so, it is appropriate to note that during the same period, client visit frequency significantly declined in the control clinics while increasing in the franchised clinics. This suggests that the GSF intervention may have motivated current users to more often keep follow up appointments and/or not visit other providers but return to the CHS with questions and other matters related to the clinic's RHFP services. It seems likely that the increased usage frequency will translate into higher word-of-mouth levels which will ultimately increase use and/or prevent future usage decline. Of course, such possibilities must await future study for confirmation.

This study also found several other determinants of service utilization at the local CHS. For example, the logistic regression analysis found that non-farmers were significantly less likely than farmers to visit their local CHS for RHFP services (OR = 0.58; *p *< .001). As farmers often have lower incomes, this finding suggests that there may be an association between income and CHS service use. Occupational groups with higher incomes may still prefer other health facilities. This finding is consistent with studies showing that lower income people use CHS services [9] and franchised health services [1] more frequently. Perhaps, higher income individuals did not respond as positively to communication activities designed to change perceptions of franchised CHS quality. Because high-income groups are often more willing to pay for quality services, they constitute an important potential client group for GSF clinics when user fees are applied. To attract new clients to CHS services, non-farming occupation groups should be targeted in the future with research-based benefits that motivate trial and ongoing use.

In addition, the logistic regression model found that ever-married individuals were more likely to use RHFP services provided at their local CHS (OR = 2.1; *p *< .01). This finding is reasonable, given the low prevalence of unmarried women in Vietnam who are sexually experienced [14]. However, because premarital sexual activity is increasing in Vietnam [14], this finding may also imply that unmarried people who are sexually active prefer RHFP services in other health facilities (e.g., private clinics, higher level hospitals). Moreover, due to social taboo and stigma associated with premarital sex in Vietnam and elsewhere, unmarried people, particularly women, often feel "shameful" when accessing RHFP services through a local health facility [11,15]. Thus, CHS services may require further adaptation to meet the needs of this segment.

Strengths of this evaluation are noteworthy. First, a quasi-experimental design with a control group selected from a neighbor province with similar socio-cultural characteristics increases the comparative evaluation's internal validity. Second, the GSF model's impact on client usage behavior was assessed using multiple outcome measures at both the individual level and clinic level. Third, with 2 follow-up surveys that had a reasonably high response rate, the time trend in service utilization was taken into account when evaluating the intervention's impact.

This evaluation also has limitations. First, usage data were not supplemented with health outcome measures, for example, reproductive tract infection rates. This prevents objective assessment of service quality. Increased client volume may have been driven by franchise membership advertising rather than improvements in service quality. Second, 12-months may not be sufficient to assess GSF model sustainability. Third, while the quasi-experimental design employed in this study generally possesses higher external validity than randomized approaches, it may suffer from threats to internal validity. For this reason, replication would increase confidence in the effectiveness of the GSF model. Finally, self-reports such as clinic visit frequency may be subject to recall biases.

## Conclusions

The study finds positive associations between GSF membership and client volumes as reported by the clinics at the end of the evaluation period. It also documents a positive relationship between GSF membership and self-reported visit frequency. Given growing interest in RHFP franchising in developing countries, this study provides preliminary evidence regarding a new approach - the Government Social Franchise model - that has tremendous potential for effective use of physical and human capital investments to improve RHFP capacity of smaller public sector clinics.

Scaling-up the GSF network will require additional funding that may be available from several sources. In the near term, foundation and foreign development assistance may be secured. In the medium term, the government may embrace the GSF model and allocate funding to the public health system to upgrade additional clinics, enabling expansion of the franchise network. Before the model can become a government priority, however, further investigations, including assessment of health outcomes associated with increased use of GSF services and cost-effectiveness of the model, are required to better delineate the effectiveness and limitations of franchising RHFP services in the public health system in Vietnam and other developing countries.

## Abbreviations

CHS: Commune Health Station; CI: Confidence Interval; GSF: Government Social Franchise; FP: Family Planning; RH: Reproductive Health; OR: Odd Ratios; Coeff: Regression Coefficient.

## Competing interests

The authors declare that they have no competing interests.

## Authors' contributions

AN and DA conceived research ideas. AN, HP, VP developed research protocol, data collection tools, and conduct data collection and data entry. AN performed data analysis and drafted the manuscript with input from HP and VP. DA made a substantial contribution in revising the manuscript for intellectual content. All authors reviewed and approved the final version.

## Pre-publication history

The pre-publication history for this paper can be accessed here:

http://www.biomedcentral.com/1472-6963/10/54/prepub
